# The Nature and Treatment of the Diseases of the Ear

**Published:** 1837-01

**Authors:** 


### ART. IV.

*Die Erkenntniss und Heilung der Ohrenkrankheiten.* Von Dr. WILHELM KRAMER.—*Berlin*, 1836.

*The Nature and Treatment of the Diseases of the Ear.* By Dr. WM. KRAMER, of Berlin. *With Copper-plates.*—*Berlin*, 1836. 8vo. pp. 400.

THIS is rather a new work than a new edition of a Memoir on Chronic Deafness, published by the author three years ago.* The earlier work was fragmentary; the present, Dr. Kramer puts forward as a complete treatise on Diseases of the Ear.

The first section contains a critical analysis of the literature of diseases of the ear, in chronological order, from the earliest times, concisely and searchingly written; but our limited space will oblige us to pass over this part of the work with slight notice. Up to very recent times there has prevailed the greatest uncertainty in the knowledge and want of plan

* Erfahrungen über die Erkenntniss und Heilung der langwierigen Schwerhörigkeit. 8vo. Berlin, 1833.

in the treatment of the diseases of the ear; and this, Dr. K. asserts, has been owing, in a particular manner, to the neglect of actual examination of the organ in its diseased state.

Hippocrates does not treat of affections of the ear as separate forms of disease. He merely mentions them as accompaniments of fevers and other acute complaints, and even then only according as they are prognostics of a favorable or unfavorable termination. Celsus, who first described affections of the ear as distinct diseases, gave excellent rules for the treatment of the more violent inflammations of this organ, and recommended ocular inspection of the auditory passage in cases of long-continued deafness. But, unfortunately, the local use of acrid stimulating remedies, recommended by him for all diseases of the ear without distinction, has obtained, even to our time, the greatest favour from practitioners. Galen adopted this practice also, although he blames Appollonius for advising pains and ulcers of the ear to be treated indifferently with the most violent stimulating substances; but, indeed, it must be confessed that, in Galen's time, the knowledge of the ear was retrograde, compared with its state in the days of Celsus. For more than a thousand years the crude empirical practice of Galen in diseases of the ear maintained full and undiminished sway. The additions made to the knowledge of the anatomy of this organ towards the end of the fifteenth and the first half of the sixteenth century, by the labours of Achillini, Vesalius, Ingrassias, Eustachius, Fallopius, Casserius, and others, exerted little influence on the pathological and therapeutical opinions of the physicians of that time; so that, in the work of Mercurialis, we do not find anything more than was given by Galen fourteen hundred years before. Fabricius Hildanus was the first who recurred to the true way of investigation, but he confined his observations to the auditory passage. He was the inventor of the *speculum auris*. The necrotomic examinations of Bonetus in reference to the ear are only to be considered as examples how such inspections ought *not* to be made, if they are intended to be of use to science. A few years after Bonetus, Duverney published a work on the ear, the anatomical part of which, abounding as it does in excellent and illustrative investigations and descriptions, has gained, even for the annexed pathological and therapeutical treatise, a meed of approbation to which it is by no means entitled. Still Duverney in that work went a step beyond his predecessors, inasmuch as he has not only considered the diseases of the auditory passage and of the membrana tympani, but also those of the cavity of the tympanum and labyrinth. But we admire in Duverney the anatomist of the ear, not the surgeon of its diseases; and in this category we must include, with still greater reason, Vieussens, Valsalva, and Cassebohm.

To a postmaster of Versailles, of the name of Guyot, we are indebted for the hint of a discovery which forms an epoch in the history of diseases of the ear; a discovery which has imparted to the diagnosis and treatment of the diseases of the middle and internal ear the only sure basis: we mean the possibility of introducing a catheter into, and consequently injecting, the Eustachian tube. Guyot, having some knowledge of anatomy, was led to think of this, with the hope of doing something for the alleviation of a deafness under which he himself laboured. For this purpose he invented a syringe, which he presented, in 1724, to the

Royal Academy of Sciences of Paris. But he proposed to introduce the catheter into the Eustachian tube by the mouth, a thing not practicable, though he might, as the reporters of the Academy remark, have succeeded in washing out the extremity of the Eustachian tube. It is probable that it was merely by this that his own deafness was relieved. For the very imperfect and indeed impracticable procedure of Guyot, Archibald Cleland, an English army surgeon, substituted, in 1731, the introduction of a flexible silver tube through the nose into the Eustachian tube. Petit had previously proposed, and Douglas had demonstrated in his anatomical lectures, the possibility of passing a probe, &c. into the Eustachian tube by the nostrils. But Wathen was the first who gave cases in which, by injections into the Eustachian tube, a partially favorable result, at least, was obtained.

We are sorry that we cannot follow our author through his interesting and instructive history and criticism of the numerous publications on diseases of the ear, which have appeared in the different countries of Europe in more recent times. There does not appear to have been hitherto much that is valuable on diseases of the ear in the medical literature of Germany. Lentin, Himly, Schubert, Trampel, Albrecht, Van Hooven, Beck, Riedel, Vering, Rauch (of St. Petersburg), Krukenberg, and Lincke, are the chief writers. Of these, the works of the two last-named are most deserving of notice. The inflammatory affections of the ear are successfully illustrated by them, but they have failed in sufficiently discriminating their different sites and forms.

On turning to the medical literature of France, we find works characterized by a profundity which we have looked for in vain in those of Germany and England. At the head of the French medical literature on the ear stand the works of Itard and Deleau, with whom neither Demonçeau nor Alard can be associated. Montfalcon is a slavish follower of Leschevin; and the posthumous work of Saissy scarcely deserves the encomiums which have been lavished upon it. Saissy writes too much in the old style of imagining diseases, and then devising modes of cure for them. The latest work on the ear published in France is a small brochure, entitled "Recherches sur la Surdité, par J. V. Gairal," which contains what the author is pleased to call a new method for the catheterism of the Eustachian tube. He describes instruments of his own for this purpose and for the perforation of the membrana tympani; but we cannot see that they are improvements. Perhaps the hint for the employment of an elastic gum bag for injecting air into the Eustachian tube may be occasionally useful, it being more readily obtained than an air-condensing machine.

In England, the state of medical science relating to the ear, and the art of the aurist generally, are in a condition vastly inferior to the same branches in France, and even in Germany before the works of Dr. Kramer. The initiatory essays at improvement by Cleland and Wathen were not followed up as they ought to have been. Sir Astley Cooper's papers in the Philosophical Transactions for the years 1800 and 1801, attracted considerable attention to the subject at that time; and we may look upon Mr. Saunders's work, emanating as it did from the school of which Sir A. Cooper has been so bright an ornament, as a result of the impulse thus given. Mr. Saunders's is not, however, a work from which we can derive any fundamental notions of the subject, and is totally deficient in data for forming a correct diagnosis. He has not even been successful in elucidating that which he made his particular object of investigation, viz. what he calls *the puriform discharge from the tympanum,* under which name, we think, he has confounded distinct diseases of the auditory passage, membrana tympani, and cavity of the tympanum. Mr. S., however, deserves credit for his attempt to improve the state of the surgery of the ear in this country; and it is to be regretted that his endeavours have not been seconded by any person qualified for the task. The practice in diseases of the ear has been almost entirely left in the hands of persons whom education had in no way fitted for scientific enquiry.

Mr. Curtis, in his treatise on the Physiology and Pathology of the Ear, has appropriated the *whole* of Mr. Saunders's essay. The exact words, indeed, have in some instances been changed, but the plagiarism is too manifest to escape even the most inattentive reader. To this paraphrase of Mr. S.'s work, Mr. Curtis has added some things from other authors, and some histories of cases treated by himself, (of course all most successfully,) and has thus concocted a treatise which, with singular effrontery, he has put forth as entirely of his own composition, and as containing the results of his own practice. This work has now, for a period of about twenty years, been forced upon the attention of the public, by the advertisement of successive editions; and it is a melancholy fact that there should have been found editors of even medical journals either so ignorant or so careless as to lavish commendation on such a production.

If we take up the works of Wright and Stevenson on the ear, with the expectation of finding anything much better in them, we shall be disappointed. Though Stevenson has not pillaged Mr. Saunders's work as Mr. Curtis has done, we yet recognize the same ideas running throughout his treatise. "Wright," says Dr. Kramer, "is only exceeded in shallowness and emptiness by Stevenson and Curtis." We may add, he is not a whit behind them in pretension.

Mr. Buchanan, a respectable and well-educated practitioner, has published some works on the ear. Dr. Kramer thinks that the only good thing in Mr. B. as an aurist is, that he practises catheterism of the Eustachian tube; but, with the exception of the "surgical remarks on introducing the probe and catheter into the Eustachian tube by the nostril," appended to the description of the "engraved representation of the anatomy of the human ear," we are not aware that his writings afford much evidence of his employing injections into the Eustachian tube as an important therapeutical agent. Mr. B.'s nosological arrangement, Dr. Kramer says, abounds in errors and repetitions.

The last English work treating of the diseases of the ear we have to notice is an essay containing "a few remarks on Congenital Deafness, on the Diseases of the Ear, and on some Imperfections of the Organs of Speech," appended to a "Treatise on the Anatomy and Physiology of the Organ of Hearing," by Mr. Tod. This gentleman devotes two long chapters to the consideration of the causes and treatment of congenital deafness, but he appears to have totally mistaken the proper object and mode of investigation. Instead of drawing inductions from carefully observed and well-established facts, he loses himself in speculations which we cannot otherwise characterize than as crude, inconclusive, and little to the purpose. The treatment proposed by Mr. T. for congenital deafness, when depending on derangement of the structure of the tympanum, is by the introduction of acrid substances, such as ammonia, tincture of cantharides, ether, the mineral acids, &c., to cause such inflammation as will be sufficient, according to him, to rouse in some degree the reproductive powers of the textures contained in that cavity. And these acrid substances are to be employed to the extent even of inducing suppuration in the cavity of the tympanum. Mr. T. gives four cases illustrative of this mode of treatment, but they are related in too indefinite a manner to convey much information; and, on the whole, we cannot say that the author has added to our knowledge of the diseases of the ear.

Before dismissing the subject of English medical literature on the ear, we ought to allude to Mr. Swan's observations; and we enter upon this notice with a feeling very different from that which influenced us in speaking of certain of the authors whose works we have been criticising. Mr. Swan, in his Treatise on the Diseases and Injuries of the Nerves, in discussing the diseases of the auditory nerves, expresses his opinion that deafness, and the noises which accompany it, very often depend on the state of the nerves distributed on the membrane lining the tympanum. "He wishes to establish that, in a great proportion of habitually deaf people, the auditory nerves are not affected." We shall hereafter state our opinion of this theory; and must now advert to another. There is at the bottom of the internal auditory meatus a communication between the auditory and facial nerves, which has been pointed out by Swan, Arnold, and perhaps by Köllner. By this communication Mr. S. endeavours to explain a circumstance which he considers he has established, viz. that in cases of deafness from some other cause than disease of the auditory nerve, sound is conveyed through the medium of the facial nerve of the seventh pair, and some other nerves connected with it. We must be cautious of admitting the conclusions of even the highest authorities, if we are not assured of their having formed a correct diagnosis by carefully instituted local examination. That Mr. Swan has not sufficiently attended to this, may be inferred from the circumstance that he speaks of a case of deafness which he thought might arise from imperforate Eustachian tube, for no other reason than because noises in the ear, which Mr. S. considers the usual characteristic symptom of nervous deafness, were wanting. He therefore performed perforation of the membrana tympani in each ear, but without any good result. Of the insufficiency of the indication and the impropriety of the operation in this case, we shall have occasion hereafter to speak.

We must now proceed to examine the practical part of Dr. Kramer's work.

"In the present treatise," says Dr. K., "it has been my endeavour to arrange the diseases of the ear in a more natural manner than has been hitherto done, to found them on decided organic alterations of the constituent structure of the ear, to avoid all hypothetical and speculative admissions in our arguments; but, above all, to deduce the diagnosis of each form of disease from attention to the actual symptoms, independent of the always doubtful accounts of the patient, and on this sure basis to establish, as far as possible, a simple and effectual method of treatment." (P. 30.)

Before entering on the consideration of individual diseases, we shall first notice what Dr. Kramer says of the pretensions of the principal remedies which have been from time to time vaunted as specifics against *Deafness*, and of the frequency and curability of diseases of the ear in general.

First on the list of Locally Acting Remedies, which have been vaunted as capable of curing deafness, come *electricity*, *galvanism*, and *mineral magnetism*, which may be all included in the same category. Dr. K. designates electricity as the least efficacious of this class of remedies. He examines into the proofs of the cases adduced by Mauduyt, Cavallo, Le Bouvier, Desmortiers, and Busch, in support of the efficacy of electricity; and he very clearly shows that not one individual could be said to have been cured, though many were made worse. Itard, speaking from his own experience, says that electricity is without any useful effect on the ear; and in this opinion Deleau perfectly coincides with him. Experience has shown that galvanism is as little to be depended on as electricity. As to mineral magnetism, its action is so nearly allied to that of electricity and galvanism, that no better effect was to be expected from it, and the result has confirmed this anticipation. Many cases, indeed, have been published as cures of deafness by mineral magnetism; but it will be found that what real improvement may have taken place was only temporary, such as has been observed to occur from galvanism, in consequence of the increased irritability excited during the employment of the agent. In most cases, however, the improvement was only apparent.

Of *moxa* and the *actual cautery*, Dr. K. remarks, that, "even on Itard's recommendation of them we can lay no weight. They are too heroical remedies to be used without having good reason to expect a beneficial result."

"*Blisters* and *tartar emetic ointment* are," says Dr. K., "only indicated in circumscribed chronic inflammation of the auditory passage and membrana tympani." He prefers tartar emetic ointment, which he rubs in below the mastoid process, to avoid all risk of injuring that part.

In regard to *issues* and *setons*, Dr. K. says, "setons are of as little value as issues. . . . All patients who have worn a seton have unanimously described the injurious influence of it on the disease of the ear."

There is no disease of the ear in which *douches*, either of water or vapour, thrown into the auditory passage, are indicated: not so, however, as will be seen hereafter, with the douches introduced into the Eustachian tube by means of the catheter.

All *drops* and *injections*, especially those of a spirituous, acrid, irritating nature, though sanctioned by long use, are pronounced by Dr. K. to be pernicious. All quack medicines for deafness are of this kind.

Of the General Remedies, Russian vapour baths have been much and indiscriminately recommended in diseases of the ear, in consequence of the supposed origin of most of them from cold. But, even supposing this to be the case, surely it is not to be expected that the local malady will be at once removed along with the general disease which may have given origin to it.

Sea-bathing has been as much recommended, especially in supposed cases of nervous deafness, but unfortunately the results have not answered the expectations.

*Warm baths, sulphur, chalybeate, and other baths,* are injurious, if there be any congestion in the head and ears; if otherwise, they are admissible only when a general complaint, existing along with the deafness, urgently requires their use.

*Emetics* are of no use in nervous deafness. They have been used for the purpose of clearing an obstructed Eustachian tube: they may have this effect if there be merely a collection of mucus closing up the mouth of this canal; but, if the obstruction from mucus be more extensive, they are nugatory.

*Purgatives* are admissible only as auxiliary remedies in acute and chronic inflammations of the ear, as of other parts; but they must be considered injurious in nervous deafness.

*Bleeding* ought only to be employed on general principles.

"The treatment by *salivation, abstinence,* and *inunction,* I believe," says Dr. K., "can never be indicated for any disease of the ear, as such."

*Arnica flowers* in infusion have been vaunted as a specific remedy for paralysis of the auditory nerve from rheumatic metastasis; for the same reason, we suppose, that many other remedies have acquired reputation in particular diseases,—viz. because they happened to be used at a time when the disease, having resisted all medicaments previously employed, began to yield of its own accord.

"If," says Dr. K., "by this exclusive criticism of the general methods of cure directed against the diseases of the ear, it should be supposed that I mean to consider them as altogether isolated, independent of all connexion with the diseases of the rest of the organism, I formally protest against such an inference. On the contrary, it is my deliberate conviction that, in every disease of the ear, especially if of long continuance, the general health of the patient must be most carefully regulated according to the rules of general and special therapeutics; not with the intention or hope, however, of improving, much less curing, in this way, the disease of the ear, which certainly will not succeed; but only in order thereby to clear and level the ground on which the superstructure of the special treatment of the disease of the ear is to be erected." (P. 89.)

After insisting on the importance and necessity of careful local examination in all cases of diseases of the ear, Dr. K. proceeds to remark on the *curability* of the diseases of the ear.

"We ought not," he says, "to allow ourselves to be imposed upon when we read that Curtis (whose inconceivable ignorance of what has been done in this department of medicine, and whose crude empiricism in the treatment of diseases of the ear we shall have often occasion to expose,) from the year 1817 to 1829 inclusive, treated in the London Dispensary for Diseases of the Ear 8,782 persons; of whom, 3,780 were dismissed perfectly cured, 2,497 improved, and only 2,505 without any improvement. In this report there is a total want of all intrinsic credibility. The same improbability exists, nearly to as great a degree, in the works of Wright. The account which he gives of the brilliant results of his practice must strike us as highly incredible, if we only consider his assertion that gargling is quite as effectual as injections in the diseases of the Eustachian tube and cavity of the tympanum! We must therefore withhold our confidence from him when he asserts that he has, out of 1500 patients, cured 496, considerably improved 380, partially 290; whilst, of those remaining, 210 either continued under treatment or had given up attendance, and only 124 had been dismissed incurable. In juxtaposition with these altogether incredible assertions, we place a table in which 300 patients have been carefully arranged according to the different forms of disease under which they laboured, and classed under different heads according to the therapeutical results obtained.

*Tabular View of the relative Frequency and Curability of Diseases of the Ear.*

| Name of the Disease. | Incurable and not treated. | Cured. | Relieved. | Uncured. | Total. | | | |
|---|---|---|---|---|---|---|---|---|
| OF THE CARTILAGE OF THE EAR. | | | | | | | | |
| Erysipelatous inflammation | ...... | 1 | ...... | ...... | 1 | 3 | 85 | In the external ear. |
| Scirrhous degeneration | ..... | 2 | ...... | ...... | 2 | | | |
| IN THE AUDITORY PASSAGE. | | | | | | | | |
| Erysipelatous inflammation | ...... | 17 | ...... | ...... | 17 | 46 | | |
| Inflammation of the glandular integument | 3 | 9 | 13 | ...... | 25 | | | |
| Inflammation of the cellular tissue | ...... | 2 | ...... | ...... | 2 | | | |
| Inflammation of the periosteum. | 2 | ...... | ...... | ...... | 2 | | | |
| OF THE MEMBRANA TYMPANI. | | | | | | | | |
| Acute inflammation | ...... | 1 | ..... | ...... | 1 | 36 | | |
| Chronic inflammation... | 11 | 7 | 17 | ...... | 35 | | | |
| IN THE CAVITY OF THE TYMPANUM AND EUSTACHIAN TUBE. | | | | | | | | |
| Inflammation of the mucous membrane, with obstruction | ..... | 28 | 6 | ...... | 34 | 55 | 55 | In the middle ear. |
| Inflammation of the mucous membrane, with stricture of the Eustachian tube | 16 | ...... | 3 | ...... | 19 | | | |
| Inflammation of the mucous membrane, with obliteration of the Eustachian tube | 1 | ...... | ...... | ...... | 1 | | | |
| Inflammation of the cellular tissue of the cavity of the tympanum | ...... | ...... | ...... | 1 | 1 | | | |
| IN THE LABYRINTH. | | | | | | | | |
| Erethitic nervous deafness | 60 | 21 | 52 | 7 | 140 | 152 | 152 | In the internal ear. |
| Torpid nervous deafness | 3 | 8 | 1 | ...... | 12 | | | |
| Deafness and dumbness | 8 | ...... | ...... | ...... | 8 | | 8 | |
| | 104 | 96 | 92 | 8 | 300 | | 300 | |
| | | 188 Relieved. | | | | | | |

Dr. Kramer commences the second section of his work with some remarks on the classification of the diseases of the ear. We have not space to enter further into the subject than to notice his own system, which is natural and comprehensive, being founded on the only sure basis, the difference in structure of the parts affected. "In all diseases of the ear," he says, "it should be our endeavour, by a careful examination of the affected organ, to determine the seat of the disease and the organic relations of the symptoms, as this is the only way by which we can attain to a knowledge of the most suitable and effectual mode of treatment in each individual case." (P. 97.)

He first divides the diseases respectively into those of the external ear, those of the middle ear, and those of the internal ear, as in the preceding table.

The diseases of the external ear are: 1. Diseases of the Auricle, comprising, *a*, erysipelatous inflammation; *b*, scirrhous degeneration; *c*, boils. 2. Diseases of the auditory passage, comprising, *a*, erysipelatous inflammation; *b*, inflammation of the glandular part of the investing integument; *c*, inflammation of the cellular tissue; *d*, inflammation of the periosteum. 3. Diseases of the membrana tympani, comprising, *a*, acute inflammation; *b*, chronic inflammation.

The diseases of the middle ear are: 1. Inflammation of the mucous membrane of the middle ear: *a*, with the formation and accumulation of mucus; *b*, with stricture of the Eustachian tube; *c*, with obliteration of the Eustachian tube. 2. Inflammation of the cellular tissue and periosteum in the cavity of the tympanum: *a*, the acute form of true internal inflammation of the ear; *b*, the chronic form of it.

The diseases of the internal ear are, the two forms of nervous deafness: *a*, with erethism or excitement; *b*, with torpor.

In presenting to our readers what to us seems most worthy of notice in the account which Dr. Kramer gives of these different diseases of the ear, our object will be to give such an abstract of the whole as will convey some idea of the scope of Dr. K.'s work, of the unsparing, but we must confess too well justified, spirit of criticism which pervades it; and of the impulse which it seems to us calculated to communicate to the study of the diseases of the ear. He has proved we think, that, contrary to the generally received opinion, the diseases of the ear are nearly as capable of accurate and fruitful investigation as are those of the eye; and we consider that he has succeeded to a considerable extent in bringing our knowledge of their nature and proper treatment to the level at which our knowledge of the eye has arrived.

We pass over altogether the account of the diseases of the auricle. To form a correct diagnosis of the diseases of the auditory passage and membrana tympani, and negatively of the diseases of the other parts of the ear, it is above all things necessary that the greatest attention should be paid to the investigation of the auditory passage by ocular inspection. To the neglect of this inspection, and to the neglect of the investigation of the state of the Eustachian tube and cavity of the tympanum by means of the catheter and injections, is to be attributed our very defective diagnosis in this class of diseases, and consequently the inefficiency, not to say injuriousness, of our treatment of them.

"In consequence of the curvature of the auditory passage, the bottom of it and the membrana tympani occupy such a position, that they cannot be seen distinctly, in the usual natural width of this canal. To effect the examination, the patient's head must be inclined strongly to the opposite side, and the ear being directed towards the sun, the auricle is drawn well upwards and outwards, whilst the tragus is at the same time pressed outwards. By this arrangement, the rays of light are allowed to fall on the parts to be examined, provided the auditory passage and membrana tympani are sound. But when morbid changes have taken place in these parts, a particular instrument is requisite, in order to remove the curvature of the passage and convert it for the time into a straight canal, so that the light may easily penetrate to the bottom. Hildanus, as was observed above, first mentioned an instrument for this purpose, under the name of *speculum auris*. But the arms of his instrument have a pyramidal shape unfavorable for the introduction of it into the auditory passage. Since Hildanus no mention of this very indispensable instrument has been made, not even by Itard, Saissy, and Deleau. Jos. Frank, indeed, speaks of a *speculum auris,* without describing it, and Wright rejects as totally deficient in utility Weiss's three-armed speculum; whilst he praises his own instrument as simple but effective, though he does not describe it." (P. 117.)

Dr. K.'s speculum, of which he gives a figure and description, differs from the one in common use in this country only in having the further end more taper, with less of the oval form, and in having a wider funnel-like orifice. The inner surface of the funnel ought to be painted black or corroded, so as to be quite dim. A polished surface, by reflecting the rays of light, confuses the examination. It is of some importance to attend to this, because a contrary opinion seems to prevail; for all the specula we have seen in this country are highly polished on the inner surface of the funnel; nay, we meet with gilded ones, in order, as we are told, that the reflexion may be greater.

"For the examination of the auditory passage," continues Dr. K., "no artificial illumination can equal, much less supersede, the light of the sun's rays. We must therefore always have recourse to them in important cases, such as operations in the vicinity of the membrana tympani. Notwithstanding, however, the failure of all attempts to find a substitute for the rays of the sun, not always at our disposal, enough has been effected to enable us during dull weather successfully to examine the more evident forms of disease." (P. 119.)

After pointing out the inefficiency of the means proposed by Cleland, Bozzini, Deleau, and Buchanan, for this purpose, Dr. K. gives us a description of an improved apparatus invented by himself, the principal part of which is an Argand's lamp; and the great mass of light from this falls upon a concave metallic mirror, whence it is reflected to a convex lens, through which, and through a second convex lens contained in the tube of the apparatus, the rays of light pass so as to be collected into an intensely bright focus, of the size of a two-groschen piece, (a shilling.)

"But whether," (says Dr. K.) "we employ the sun's light or artificial light, the speculum ought always to be had recourse to in important cases. Its assistance is especially necessary to enable us to accomplish the examination with the eye alone, without being obliged, like Itard, Curtis, Wright, Buchanan, Tod, and others, to use the probe. This is improper, partly because it affords no correct diagnosis, but chiefly in consequence of the great delicacy and sensibility of the membrana tympani." (P. 121.)

The number of all diseases has been much multiplied by the practice of considering the different effects of some primary diseases, such as inflammation, nay even certain symptoms, as so many separate and independent forms. The result of this has been great confusion in the whole range of pathology, but perhaps there are no organs in which the practice has been carried to a more vicious extent than in the case of the eye and the ear. "Careful observation," says Dr. K., "has taught me that all the different forms of disease which affect the auditory passage depend on inflammation of its constituent structures, and they are very characteristically defined, according as one or other structure is affected. The effects of these forms of inflammation have no claim to be considered as separate forms of disease, but naturally come under that which has given rise to them." (P. 125.) On this principle the diseases of the auditory passage really to be distinguished, are those already mentioned, viz. erysipelatous inflammation; inflammation of the glandular part of the investing integument; inflammation of the cellular tissue, and inflammation of the periosteum.

Those great accumulations of hard, dark-brown wax in the auditory passage, which so often produce deafness, are the result of erysipelatous inflammation. "This accumulation of ear-wax," says Dr. K., "has been very unjustly laid to the charge of neglect and want of cleanliness on the part of the patient. It is itself a diseased product, the removal of which no patient can effect, as the naturally very sensitive auditory passage becomes still more so in consequence of the erysipelatous inflammation, and even at its anterior part cannot endure the slightest touch." (P. 129.)

Mr. Buchanan, in his "Illustrations," devotes a chapter to the subject of "Syringing the Meatus." He insists very much on the advantage of employing a syringe with a slender point, in order that there may be a counter-current of the injection, which will force all loose extraneous bodies outwards. He also recommends that the syringe should be no larger than to contain three drachms of injection. He fears that, with a syringe having a larger point, and containing more water, "there will be danger of rupturing the membrana tympani, and dislocating the ossicula auditus, from the quantity of liquid thrown into the meatus, and the counter-current of the injection being obstructed by the point of the syringe."

"All this," Dr. K. remarks, "is useless and unnecessary precaution. The water flows out very well in spite of the thick pipe, and brings away with it the loosened ear-wax. No stream of water from a strong ear-syringe can injure the membrana tympani, particularly as the injection is not directed upon it, but upon the hardened ear-wax. The small syringes usually employed for this purpose, are therefore worse than useless, because they contain too little water, protract the operation to an indefinite extent, and, in consequence of their long pipe, there is danger, in restless patients, of pushing it too deep into the passage and causing unnecessary pain, if not injury of the membrana tympani. I therefore use an ear-syringe which is three inches long, contains an ounce and a half of water, and is furnished anteriorly with a pipe three quarters of an inch long, and with an opening wide enough to give passage to a strong stream of water." (P. 132.)

As to the fluid used for the injection, Dr. K. says that any thing but tepid water is perfectly superfluous. He has never seen a case of hardened ear-wax, in which, with tepid water, it was not syringed out in half an hour. (P. 133.)

Inflammation of the glandular part of the investing integument of the auditory passage is what is commonly known under the name of *catarrhal* inflammation of the external ear. From this inflammation, and from that of the membrana tympani, the polypous growths of the auditory passage derive their origin.

"By vascular inspection," says Dr. K., "we discover (in the inflammation we are at present considering) on the walls of the auditory passage, inflammatory redness, and partial swelling, which when it attains a farther degree of development and greater elevation, receives the name of a fleshy excrescence or polypus. These excrescences are either soft, spongy, of a bright red colour, vesicular, bleeding on every touch, sensitive, covered with a copious mucous secretion, pedunculated, globular, or they have a broad basis, almost as hard as cartilage or even as bone, insensible, bleeding little or not at all, and rather of a pale red colour. The inflammatory origin of these polypi has been unjustly called in question." (P. 141.)

Collections of thickened, hardened ear-wax are considered, among many other things, as causes of this catarrhal inflammation; but Dr. K. says (p. 145,) that repeated experience has decidedly convinced him "that even a very long continuance of hardened ear-wax in the auditory passage cannot affect the glandular integument.

"Inflammation of the cellular tissue in the auditory passage, or *phlegmonous inflammation,* is distinguished," says Dr. K., "from the inflammation of the glandular integument by the abscess which always takes place in the former, while such a termination is altogether foreign to the latter. By its rapid course, and the absence, of examination with the probe, of a curious bony surface, this inflammation is just as decidedly distinguished from the inflammation of the periosteum. It might be more readily confounded with internal inflammation of the ear, which comes on with even more violent symptoms; but there is always this diagnostic mark that the internal inflammation of the ear, at least at its commencement, leaves the auditory passage perfectly free. This phlegmonous inflammation is usually confounded by authors, under the general appellation of *otitis externa,* with the slight catarrhal form of the inflammation of the glandular integument." (P. 172.)

Phlegmonous inflammation of the auditory passage is rather a rare form of disease; it is generally produced by cold.

Inflammation of the periosteum of the auditory passage (*metastatic inflammation,*) is always attended by caries, and, when any exfoliation of the diseased bone takes place, the ulcerated part begins to heal, but obliteration of the passage, to a greater or less extent, is very apt to take place. As in all analogous cases, the part, even when opened up by art, has a constant tendency to grow together again.

"Even in the most favorable cases," says Dr. K., "it is difficult, not so much to open up the parts which are grown together as to maintain them open." . . . . . . We always succeed best by touching the parts with lunar caustic, which, by means of a slender caustic-holder, should be carried through and through the part which has been opened up, in its whole length." . . . "Even after cicatrization of the separated parts, hearing remains very dull, partly because the operation does not restore the natural form to the auditory passage, and partly because the membrana tympani has always suffered from the preceding inflammation, having become thickened." (P. 181.)

*Diseases of the Membrana Tympani.* "The very concealed situation of this membrane," says Dr. K., "has for a long time kept up the most erroneous hypothetical opinions of its morbid states. Under their influence we at one time looked upon a separate diseased state of the membrana tympani as impossible." . . . . "Objective symptoms," he continues, "ought alone to decide as to the diseased state of the membrana tympani, particularly as it is accessible to investigation by our senses in its whole extent. We can observe distinctly whether it is shining or is dim; whether it is transparent or opaque, and this, again, whether partially or in its whole extent; whether it presents its depression, or whether, in consequence of thickening of the membrane, this has become indistinct, or is altogether obliterated, &c." (P. 184.)

Much has been said of a too great relaxation of the membrana tympani: and Saissy supposes that this might be produced by destruction of the tensor tympani muscle by suppuration; while Beck thinks that, by violent sneezing, the tendon of the tensor tympani might be ruptured. All this is hypothetical; and just as groundless and fanciful is the admission of a too great tension of the membrana tympani.

Cleland supposes it probable that, by a violent clap of thunder, noise of a cannon or the like, the position of the membrana tympani may be altered, being forced inwards upon the small bones, and so rendered concave outwardly. The means by which Cleland proposes to remedy this supposed disorder are: first, to oblige the patient to stop his mouth and nose, and force the air through the Eustachian tube into the barrel of the ear by several strong impulses, which will probably push the membrane back to its natural state. But, in case of an obstruction of the Eustachian tube, the second method proposed is to introduce into the meatus auditorius externus, an ivory tube, as near to the drum as can be done, and so exactly fitted that no air can go in or out. The surgeon then taking the farther small end in his mouth, draws out by suction what air is contained in the auditory passage, by which means the membrana tympani, says Cleland, will be drawn back to its natural state, and thus the person will hear as before. This silly speculation of Cleland, which is quoted by Mr. Curtis in his "Essay on the Deaf and Dumb," p. 94, is mistaken by Dr. Kramer for Mr. Curtis's own. Dr. K. therefrom takes occasion to accuse Mr. C. of gross ignorance, for not knowing that the externally concave form is the natural state of the membrana tympani. With what justice soever Mr. Curtis may be accused of ignorance and pretension in other respects, the charge falls from him in this particular instance, unless we consider his designating the part of Cleland's paper which he quotes, "as containing some ingenious remarks on the construction of instruments proposed to remedy some kinds of deafness proceeding from obstructions in the external and internal auditory passages," as in approbation of all that is therein contained.

Dr. K. denies the possibility of the membrana tympani being torn without previous inflammation, in contradiction to the assertions of Duverney, Leschevin, Itard, Saissy, and Curtis.

"It is true," he says, "that we meet with perforations of the membrana tympani, without the discharge of a mucous or purulent fluid ; but, even in such a case, a viscid, mucous, puriform matter in small quantity is always found at the bottom, and the membrana tympani, so far as it is not yet destroyed, reddened, thickened, and opaque. We arrive at such certain results only by means of a close inspection of the auditory passage in bright sunshine, and with the assistance of a speculum; whilst, with the probe, which the above-named practitioners make use of, we never could discover such morbid changes."

In regard to the degree of hearing which may exist along with a perforated membrana tympani, Dr. K. makes the following just observations:

"Many authors, and among them even Itard, are still of opinion, that perforation of the membrana tympani does not necessarily weaken the hearing, an error occasioned by their having judged of the hearing only by the way the patient could carry on a conversation, instead of employing, as a standard of measurement, some determinate sound. Repeated careful observation of such patients as had their membrana tympani perforated, has satisfied me that this morbid state has not indeed for its consequence total deafness, but undoubtedly a greater or less degree of dulness of hearing, according to the extent of the destruction of the membrane, according as it has taken place before or behind the handle of the malleus, and according as it is solely confined to the membrane, or is accompanied by the loss of the small bones or other morbid changes in the ear. I have seen patients who could hear my watch indeed at the distance of five or six feet, but yet not at the distance of thirty feet as a sound ear can. With this slight degree of deafness they were on the whole not much incommoded, although there were to be seen in the membrana tympani holes the size of a pea; whilst other patients could scarcely hear the same watch at the distance of half an inch. In such cases as the latter, however, there must have been, besides the perforation, other morbid changes in the cavity of the tympanum, &c." (P. 192.)

The diseases of the membrana tympani which Dr. K. admits as established are only the inflammatory diseases with their consequences, as opacity, thickening, perforation, purulent secretion, and formation of polypi. In acute inflammation, the membrane is found, on examination, to be blood-red, swollen, rough as if it were covered with small glands, somewhat projecting, and opaque. Bundles of blood-vessels are seen ramifying in it, and the point of insertion of the handle of the malleus can no longer be distinguished.

"The inflammatory character of this form of disease," says Dr. K., "especially in the milder cases, has been hitherto much overlooked, and, under the name of earach, it has been subjected to the most improper local stimulating treatment. Itard indeed speaks of a purely nervous earach, in which his dread of the local use of opium had been quite groundless, if his so-called nervous earach were any thing else but an inflammation of the membrana tympani. This, to be sure, is made worse by opium, and would certainly have been recognized by him as inflammation, if he had not neglected the local investigation of the membrana tympani. . . I have certainly never observed earach without inflammatory appearances, either in the auditory passage or in the membrana tympani; and therefore, to every one who does not understand how to make, and is not in the habit of making, the examination of the membrana tympani with the speculum, and in bright sunshine, I would deny the right to pronounce an opinion as to the existence of a *nervous otalgia*. . . . Inflammation of the membrana tympani is distinguished from internal inflammation of the ear not only by its greater mildness, but especially by the morbid changes of the membrane perceptible from the very commencement of the disease. Any such morbid appearances are at first always wanting in internal inflammation of the ear, notwithstanding the most violent feverish symptoms; and, in the farther course of the malady, they occur only when the membrane is threatened with bursting by the accumulated matter, and has also become involved in the inflammatory process." (P. 195-6.)

Inflammation of the membrana tympani seems to be as little known to surgeons from actual examination, as certain of the internal inflammations of the eye, such as inflammation of the capsule of the crystalline lens. And, indeed, we need not be surprised that the diseases of an organ so difficult of access to investigation by our senses as the ear, should be so little known, when the affections of the eye, an organ so conveniently placed for our examination, are yet far from being universally understood by surgeons as they ought.

The acute form of inflammation of the membrana tympani is much less frequent than the chronic form, and the latter much more commonly leaves behind it other diseases.

Hardened ear-wax, according to Dr. K., has no more share in the production of inflammation of the membrana tympani than it has been already shown to possess in causing inflammation of the glandular integument of the auditory passage. "Sometimes, indeed," he says, "after the removal of the ear-wax, blood-vessels are observed running along the handle of the malleus, and terminating at its head; but they always disappear in a very short time without any assistance from art." (P. 197.)

The effects of inflammation of the membrana tympani are, opacity, thickening, hardening, perforation, polypi, &c.; all of which permanently injure hearing.

We are sorry to be under the necessity of greatly abridging Dr. Kramer's very excellent and interesting discussion respecting the perforation of the membrana tympani. It is not long since this subject attracted much attention, and it is well known to our readers that it was for a memoir on it that Sir Astley Cooper had the Copley Medal awarded to him by the Royal Society. "Indeed," says Dr. K., "so great was the interest which the operation excited when first proposed, that it degenerated into a sort of mania. It was thought that in it was discovered an effectual remedy against deafness of every kind, and even against deafness and dumbness." Even now, we believe, the true indications of the operation are very imperfectly understood by surgeons in general.

"If the membrana tympani," says Dr. K., "be considerably thickened, quite insensible to the touch of the probe, hard as cartilage, and if the hearing has suffered considerably, there remains nothing to be done for its improvement but the perforation of the membrane. But, even in this case, the only one which really indicates the operation, we ought to have recourse to it only when both ears are affected in the same manner with a considerable degree of deafness, or when the second ear, not having the membrane indeed diseased, yet suffers from an incurable deafness. We must, however, even in this case, satisfy ourselves by the most careful investigation that the ear to be operated upon does not suffer from any other disease, by which the result of the operation might be rendered negative.' . . . "Sir Astley Cooper supposed that the perforation of the membrana tympani was indicated principally in cases of obstruction of the Eustachian tube, and in extravasation of blood in the cavity of the tympanum; but, as he does not appear to have been acquainted with the catheterism of the Eustachian tube, his diagnosis of the closure of it was altogether uncertain. . . But, suppose obstruction of the Eustachian tube and extravasation of blood in the cavity of the tympanum really existed, these morbid states might be treated much more certainly and efficiently by the introduction of the catheter into the tube itself, than by the perforation of the membrane." . . . . "The success related by Cooper as attending his operations in several patients ought not to lead us to a favorable conclusion regarding the operation, because the diagnosis was defective, and the respective cases too indeterminately characterised. . . But as he has evidently had no experience on the subject, his opinions carry no weight with them.

"As regards the indication for the operation, Itard falls into the same error as his predecessors. If he declares somewhat more decidedly than they that the operation is admissible only when there is an *invincible* obstruction in the Eustachian tube, he yet errs, inasmuch as he does not examine whether the obstruction might not really be capable of removal. The single case in which, for thickening merely of the membrana tympani, Itard was induced to perform the operation, was followed by a favorable result, exciting to farther attempts in similar cases. Saissy recommends perforation only in hardening and thickening of the membrane; but in reference to it he does not once allude to the Eustachian tube, and cavity of the tympanum. The only case in which he has performed the operation with success is so incidentally mentioned that little value can be put upon it.

"Deleau has discussed this operation very fully in a monograph on the subject, in which he expresses his opinion that the operation may be performed with advantage in thickening of the membrane, in obstruction and obliteration of the Eustachian tube, and in obstructions of the cavity of the tympanum." . . "But his indications for operating are not more satisfactory than those of his predecessors; and he did not take pains to obtain an accurate diagnosis by a thorough examination of the Eustachian tube. It is accordingly not surprising that he should not have obtained any permanently good result worthy of being named in all the twenty-five cases operated on by him. . . The subject has been handled not less superficially by all other authors who have occupied themselves with it, either theoretically or practically. We must therefore repeat that, with the exception of a single successful case recorded by Itard, *no other* is known in which the operator was authorised in perforating the membrana tympani. We also repeat that the only thing which really indicates the operation is merely thickening of the membrana tympani, unaccompanied by any other disease of the ear. . . If it should be had recourse to in these very few excepted cases—and of these the diagnosis ought to be very carefully formed,—we must adopt a method by which the complete removal of a piece of the membrane will be effected." (P. 202, *et seq.*)

Solutions of the acetate of lead are much recommended and employed by Dr. K. in the affections of the auditory passage and membrana tympani.

"In chronic inflammation of the membrana tympani, whether with or without perforation, I employ, with the greatest benefit, a solution of the acetate of lead. It is poured, either cool or lukewarm, into the diseased ear two or three times a day. We may, according to circumstances, raise the strength of the solution from one grain to ten of the salt to one ounce of water: in which latter case the membrana tympani will be covered by the fine powder of the salt of lead, and the action of it kept up so much the longer." (P. 223.)

We doubt very much that by this means the action of the salt will be kept up so much the longer, as what is deposited is merely an inert oxide. Moreover, we suspect that opacity of the membrana tympani will frequently result from such practice, especially if there be any abrasion of the surface of the membrane, in the same manner as we find takes place in the eye on the employment of a wash containing the acetate of lead. If there be any ulcer of the cornea, or any abrasion of the conjunctiva, oxide of lead is deposited on the part, and forms a white speck.

"Instead of the acetate of lead," says Dr. K., "the nitrate of silver, the sulphate of zinc, alum, &c., in solution, have likewise been recommended; but, in my trials of them, these remedies, as well as the pyroligneous acid, in the proportion of one scruple to water one ounce, have always excited painful irritation in the auditory passage, when used in a strong solution; whilst, in a weaker, they had no effect at all. The acetate of lead removes very quickly and effectually the disagreeable ammoniacal odour of the discharge." (P. 224.)

*Diseases of the Middle Ear.* "We comprehend under this term only those diseases which are developed in the cavity of the tympanum and in the Eustachian tube, and which are accessible during the lifetime of the patient, to our diagnosis at least, if not also to our therapeutical agents." . . "Only inflammation of the mucous membrane of the Eustachian tube and of the cavity of the tympanum, with its different terminations and consequences, as well as of the cellular tissue lying under every mucous membrane, can in reality be put down as distinctly marked forms of disease. These are therefore alone considered here. (P. 240.)

Inflammation of the mucous membrane of the middle ear is described under different names by Alard, Itard, Saissy, and Deleau; but, by Curtis, Wright, Buchanan, Jos. Frank, and all other writers, it is either totally neglected or discussed only as a simple mechanical obstruction of the Eustachian tube. As the Eustachian tube forms one of the most important objects of investigation and treatment to the surgeon, we shall make pretty copious extracts from that part of Dr. Kramer's work relating to it; more particularly as, with the exception of Deleau, no other writer appears to possess the same practical acquaintance with it.

The tubes which Dr. K. recommends for introduction into the Eustachian canal are the same as those which were employed by the Montpellier surgeons, as well as Sabatier and Itard. They are made of silver, inflexible, six inches long, and of a thickness varying from that of a crow-quill to that of a goose-quill, straight except at the anterior extremity, which is bent, for the length of about half an inch, at an angle of 144 degrees, corresponding to the lateral situation of the mouth of the Eustachian tube. The tubes, or catheters, are in their whole length of the same caliber. At their posterior or nearer extremity, they are furnished with a funnel-shaped dilatation, half an inch long, for the purpose of receiving the tube of the injecting syringe, &c. At this dilatation, and in the same horizontal direction with the beak of the catheter, there is a ring soldered to, according to the direction of which we can judge of the position of the beak, when this is introduced into the nose and out of view. The tube is moreover graduated with inches, which is of great advantage in the repeated introduction of it. We pass over Dr. K.'s account of the mode of introducing the catheter into the Eustachian tube, and also the contrivances of Itard, Deleau, and himself, for retaining it in the Eustachian tube during the process of injecting.

"Through the catheter thus fixed," says Dr. K., "lukewarm water was injected into the Eustachian tube and cavity of the tympanum by Wathen, Douglas, Saissy, Itard, and others, and they supposed they could judge of the state of the middle ear according to the different sensations thereby produced in the ear, or according to the total absence of each sensation. But these aqueous injections are attended with great difficulties and defects, of which I have sufficiently convinced myself by multiplied experience.

Having stated his reasons for rejecting the aqueous injections, Dr. K. proceeds—

"All these weighty objections led Deleau to the happy idea of employing air instead of water for the investigation and treatment of the diseases of the middle ear, by which he has completely attained his object." (P. 250.)

We do not mean to advance any positive claim for Cleland in this matter, but the following quotation from his paper in the Philosophical Transactions looks very much like a suggestion of the principle:

"The pipes of the syringe," says Cleland, "are made small, of silver, to admit of bending them as occasion offers, and for the most part resemble small catheters: they are mounted with a sheep's ureter, the other end of which is fixed to an ivory pipe, which is fitted to a syringe, whereby warm water may be injected; or they will admit to blow into the Eustachian tube, and so force the air into the barrel of the ear, and dilate the tube sufficiently for the discharge of the excrementitious matter that may be lodged there." (*Phil. Trans.*)

In order to perform this operation, the air was compressed by Deleau in an apparatus, the construction of which he has hitherto kept secret. Dr. Kramer says, however, that it is very easy to construct such an apparatus; and accordingly describes and figures one, of his own contrivance, in the present work.

After declaring that the objections raised by Deleau against the inflexible silver catheter are altogether groundless, and that the elastic ones recommended by him do not deserve the preference Deleau claims for them, Dr. K. comments very severely on the hasty assertion of this author, that the elastic catheter may be pushed into the cavity of the tympanum, and employed as a dilating instrument in contraction of the Eustachian tube. "We here refer," says Dr. K., "to what has been said above of the diameter of the Eustachian tube, which, in its narrowest part, even in the sound state, does not admit the finest elastic catheter, much less in the state of stricture." It is not Deleau only he criticises, but animadverts also on Magendie and Percy, the reporters on Deleau's work to the Institute, for allowing such an assertion to pass without censure.

"After the catheter has been introduced into the Eustachian tube, and fixed by means of the frontlet, if the air-douche is to be employed for the investigation of the middle ear, the patient is placed close to a table, on which he leans the elbow next to it, and in this position he holds with the hand of that side the pipe of the air-press, previously filled with compressed air. The operator then introduces the metal beak of the pipe into the funnel-shaped dilatation of the catheter, applies his ear close to that which is under examination, opens the cock of the machine, and listens to the sound made by the air rushing into the middle ear." . . "When the Eustachian tube and cavity of the tympanum are perfectly free and open, the air flowing in strikes without interruption and with an audible shock against the membrana tympani. When the first shock of so strong a stream of air is over, or, if it was not very violent, we hear, during the continuance of the streaming in of the air, a blowing and rustling in the ear of the patient, which appears to issue out of the auditory passage, and to fill his ear in its whole extent. All variations from this sound, the peculiarities of which can only become perceptible by often-repeated observation, are morbid, and lead to very certain conclusions as to the particular diseased changes in the organization and function of the ear." (P. 257.)

The above extract shows that auscultation is not neglected as a means of obtaining information regarding the state of the ear; and it is the only instance we recollect in which the sounds to be ausculted by the application of the ear or stethoscope are produced artificially. Laennec, we believe, attempted to derive aid for the diagnosis of diseases of the internal ear by listening to the sounds produced, or which he expected might be produced, by the entrance of air naturally into it; but he was unacquainted with the method just noticed, which affords a pleasing proof of the great advance of a method of exploration which, on its first discovery, was ridiculed by a large proportion of the profession in all countries. We think it quite possible that the diagnosis of the diseases of other cavities—e. g. the intestinal canal and bladder,—may also be improved by the auscultation of sounds resulting from the artificial introduction of fluids into them. M. Deleau also speaks of a *bruit sec de la caisse*, and a *bruit muqueux*.

If the air-douche does not penetrate to the membrana tympani, Dr. K. makes use of catgut bougies for opening up the passage. He gives directions for their introduction, which we cannot stop to notice.

In treating of inflammation of the mucous membrane of the middle ear, with accumulation of mucus in it, Dr. K. has the following remarks:

"It is surprising enough, considering the great narrowness of the Eustachian tube, and the frequency of catarrhal complaints in the nose and throat, that the obstruction of the Eustachian tube with mucus is yet on the whole so rare. It certainly occurs much more frequently in moist seasons and in damp climates; as, for instance, in cities on the sea-coast, from which (as Hamburg, Stettin, Swinemünde, Danzig, Memel, Cüstrin, &c.) the greater number of patients of this kind come to me. This being the case, it is astonishing that the English (so-called) Ear-surgeons, to whom this disease must occur very often in the foggy climate of London, have scarcely an idea of the proper diagnosis of it, much less of a rational mode of treatment for it." (P. 266.)

Dr. Kramer strongly disapproves of the attempts which have been made to remove obstructions of the Eustachian tube by sending in injections through a perforation in the mastoid process, or through a perforation in the membrana tympani; and asserts, that the only safe mode of procedure is by a direct action on the guttural orifice of the Eustachian tube, and from thence on the collections of mucus found in it.

"Wathen, Douglas, Saissy, Itard, and also Deleau at the commencement of his practice," continues Dr. K., "employed aqueous injections into the Eustachian tube, which however Deleau afterwards gave up, and confined himself exclusively to the air-douche in the treatment of this disease. I have also for some years employed the water douche with great advantage; and I still consider it very appropriate, and cannot join in the excessive objections (some imaginary) brought against it by Deleau. . . Notwithstanding the undeniably good effect of aqueous injections, I have, however, of late given the preference to the air-douche, on account of the extraordinary facility, convenience, and cleanliness with which it may be managed." (P. 274.)

Inflammation with thickening of the mucous membrane of the Eustachian tube, producing stricture, has been found incurable in the hands of Dr. K.; nor has he found perforation of the membrana tympani of any use in such cases, as the mucous membrane of the cavity of the tympanum is also implicated in the chronic inflammation. The introduction of catgut bougies into the Eustachian tube has not been followed by any permanent benefit. Obliteration of the Eustachian tube must be considered, for the same reason, as quite incurable.

"Saunders and Itard," says Dr. K., "had hopes in this case from perforation of the membrana tympani. The cases which the latter communicates, however, although they have presented a favorable result, prove nothing for the applicability of the operation in obliteration of the Eustachian tube, because such a state of the latter was not in a single instance diagnostically determined by Itard. In my opinion, perforation is in this case in particular to be totally rejected, because the inflammation which has disorganized the mucous membrane of the Eustachian tube to so considerable an extent, cannot have let the mucous membrane of the cavity of the tympanum escape; whence the result is rendered more than doubtful." (P. 307.)

Inflammation of the cellular tissue and periosteum in the cavity of the tympanum, known under the name of *true internal inflammation of the ear*, presents, according to Dr. K., two forms, the acute and chronic. This is a very dangerous disease, as it may involve not only the whole ear, but also extend its ravages to the membranes of the brain.

"Krukenberg," says Dr. K., "describes under the too general name 'internal inflammation of the ear,' (to which name the catarrhal inflammation of the mucous membrane of the cavity of the tympanum has likewise a just title,) the acute form; the proper phlegmonous inflammation of the cellular tissue in the cavity of the tympanum. It is only to be regretted that he and his contemporary Abercrombie, to whom we are indebted for an excellent description of the chronic form of this inflammation, have examined in their patients neither the external auditory passage nor the Eustachian tube."* (P. 317.)

*Diseases of the Internal Ear.* As we cannot make any direct observations on the internal ear, we must, in forming our diagnosis, join to the subjective symptoms negative objective symptoms: that is, we must observe whether or not there be any deviations from the natural state of the other parts of the ear sufficient to account for the diminished hearing.

After rejecting the various hypothetical diseases of the internal ear which have been admitted by authors, apparently only for the sake of fixing on some one part as the seat of a disease they were quite ignorant of, Dr. K. adds,

"The only undoubted form of disease of the labyrinth,—that is, of the nervous

* "Abercrombie," Dr. K. adds, "diminishes the value of his observations by considering the cerebral symptoms as primary, although this view may be easily shown to be incorrect from his own cases." This is a mistake of Dr. K., as Dr. A. maintains just the opposite opinion. See Edinb. Journ. vol. xiv. p. 301; and Treatise on Diseases of the Brain, p. 33, 2d Ed. 1829.

expansions contained in it,—is functional derangement of it, under the form of changed manifestation of activity; in other words, *nervous deafness*. In this affection we have a changed, a weakened power of hearing, without any organic deviations in the whole of the ear. The term *nervous deafness* has been hitherto often enough misused as a cloak for ignorance in any doubtful, obscure disease of the ear; and it has become so suspected, that we might now be disposed rather to run into the opposite extreme, and deny its existence altogether. But this would be equally wrong. The disease certainly exists; although we may doubt the capacity of many who have used the term to apply it rightly. For, as the absence of every organic change in the ear constitutes the principal condition of purely nervous deafness, we cannot accord the right to decide on its existence to those who do not understand how to investigate the ear, and especially the middle ear, by the catheterism of the Eustachian tube. For this reason, no confidence is to be placed in any of the English so-called aurists, Curtis, Stevenson, Wright, &c.; to whom may be added the other writers on the diseases of the ear,—Saunders, Swan, Lentin, Beck, Vering, Jos. Frank, and others; even Saissy not excepted. Itard and Deleau alone, by their expertness in the use of the catheter, form an honorable exception in this case." (P. 332.)

Dr. K. admits two forms of nervous deafness; the one attended by erethism or excitement, the other with torpor. Noise in the ear forms the essential point of difference between the two. The noise in the ear, without exception, belongs to the erethitic form, whilst it is quite foreign to that characterized by torpor. But the noises in the ear are not to be looked upon as a characteristic symptom in any degree conjoined with nervous deafness only, for they accompany many other diseases of the ear. "It follows," says Dr. K., "that noise in the ear is not of itself a disease, but accompanies the most different diseases of the ear, and that often in a very indeterminate and inconstant manner. Swan, Saunders, Curtis, and others have especially fallen into this error, which has proved the more prejudicial as, on their authority, many patients, merely for the reason that they were troubled with noises in the ear, have been looked upon as affected with nervous deafness, and treated accordingly." We may add, and patients, merely because their deafness was not attended by noises, have had the membrane of the tympanum in each ear perforated.

For establishing the diagnosis of nervous deafness, we need scarcely remark, after what has been said regarding the examination of the ear, that Dr. K. insists on the most minute local investigation of all parts of the ear, and on the use of the air-douche as a means of exploration.

"In the treatment as in the diagnosis," says Dr. K,, "the English surgeons have deviated the farthest from the right path, although they affirm they have obtained by their method brilliant results. Cleland's cautious declaration, 'as for the diseases that are called *nervous*, I must leave them to the learned gentlemen of the Faculty,' has found no echo among his countrymen. With unheard-of audacity, Curtis recommends, in *doubtful* cases of nervous deafness, purgatives, especially calomel, as long as the strength permits. In decided nervous deafness, that is, in such cases as Curtis, with his very defective knowledge of ear-diseases, considers as nervous deafness, he recommends blisters, antiphlogistic diet, calomel again, and sulphate of magnesia." (P. 350.)

Dr. K. likewise condemns, although in gentler terms, the remedies advised by Swan, Saunders, Wright, and Buchanan, as being "all discordant to the true character of nervous deafness;" and "nearly quite as objectionable, he says, are the methods which are recommended by Beck, Vering, Jos. Frank, Saissy, and others."

Deleau abstains altogether from the treatment of persons affected with nervous deafness. He has not even ventured to tread in the path which Itard had indeed struck out for the rational treatment of this form of disease, but which, after a few timid steps, he relinquished.

Dr. K. rejects the two cases given by Itard as examples of the idiopathic paralysis of the auditory nerve, conceiving them to be founded on uncertain paralysis, and boldly and confidently declares "that there is not to be found in any work hitherto a single case of nervous deafness founded on an exact and carefully made diagnosis, and that as yet there has existed no proper treatment of nervous deafness."

Dr. K. makes known a mode of treatment for nervous deafness, which, if we are to judge by the successful cases he adduces and his own recommendations, we must acknowledge to be one of the greatest accessions to the therapeutics of the ear which has been made since the catheterism of the Eustachian tube. Though his mode of treatment is strictly local, Dr. K. does not neglect the general state of the constitution, strengthening the nervous system when weak, improving the digestion, regulating the functions of the bowels and uterus, and not even overlooking the condition of the intellectual functions. "But we must not flatter ourselves," he says, "that, by fulfilling these general indications, we shall improve the local affection of the auditory nerve, even in the slightest degree. . . . When the health is altogether unimpaired, as is frequently the case, we must, without delay, have recourse to the local treatment of the diseased auditory nerve." (P. 353.)

This local treatment consists in the introduction of the vapour of acetous ether into the cavity of the tympanum, through a catheter introduced into the Eustachian tube. The vapour is generated, in a proper apparatus, at the common summer temperature. Itard formerly attempted this mode of treatment, but he generated the vapour at too high a temperature; so that, instead of the simple vapour of the acetous ether, an acrid kind of gas was introduced into the ear. This gas acts very injuriously in cases of erethitic nervous deafness, although, according to Dr. K., it is the best adapted for torpid nervous deafness.

In illustration of the good effects of this mode of treatment, Dr. K. gives us ten cases, one of which only our limits will allow us to quote here.

"Case. Miss M. Wolff, eleven years old, in other respects quite healthy, had been affected, without any apparent cause, for a long time, with dulness of hearing and buzzing in both ears, for which no remedies had hitherto been employed. Both the external auditory passages, and also the Eustachian tubes, were found quite healthy. Injections of tepid water into the middle ear excited severe pain, which abated only late in the evening. With the left ear she could hear the watch at a distance of six inches, and with the right ear at a distance of two inches only. In January, 1832, ethereal vapours were for the first time introduced daily, alternately into the left and right middle ear; and no other remedy was employed at the same time. At the end of the first four weeks the buzzing in the ear completely stopped, and only returned again for a very short time in consequence of a violent exertion of the body. The treatment was continued without interruption for four months. The patient remained perfectly well in health; she had no occasion to make the slightest alteration in her usual mode of life; and, at the end of that time, the hearing distance on the left side was increased from six inches to eight feet, and on the right side from two inches to six feet. A residence in the country interrupted the treatment for nine months, in the course of which time the improvement which had been gained did not undergo the slightest diminution. In January, 1833, the same mode of treatment was renewed, and was continued (with progressive improvement of the hearing distance,) for the space of five months : at the end of that time the patient could hear the watch at fully thirty feet distance, and might therefore be considered as quite cured. The buzzing in the ears had not again occurred." (P. 361.)

It is to be wished that this mode of treatment, with all the necessary precautions as to diagnosis, manipulation, &c., were put in practice in this country by competent surgeons. How suspicious soever one might feel inclined to be of the great efficacy attributed to it by Dr. K., in some degree its discoverer, the plain statement of the facts which he brings forward forces us to admit that it is a mode of treatment of the highest value.

We remarked in a preceding page that Mr. Swan wishes to establish that, in a great proportion of habitually deaf people, the auditory nerves are not affected. He thinks that, in many such cases, the deafness is owing to a thickened state of the lining membrane of the cavity of the tympanum, involving the small branches of the tympanine nerve. We are of opinion that this theory of Mr. Swan's accords very well with the good effects derived from Dr. Kramer's practice in nervous deafness just noticed; as it appears probable that the acetous vapour may act directly on the tympanine nerves.

The fourth chapter of the second section of Dr. K.'s treatise is devoted to the consideration of Ear-trumpets; and the fifth and concluding chapter to Deaf-dumbness. In the latter chapter, Dr. K. enters into a critical examination of all the cases which have been published from time to time as cases of cured deaf-dumbness, and sums up the whole by the following declaration: "After this complete examination of all the accounts of cured deaf and dumb, we dare decidedly assert that, as yet, no individual deaf and dumb person has been really cured; that is, has been brought into such a state that he, as in the case of a sound hearing person, could, by means of the sense of hearing, hold uninterrupted conversation with his fellows under all circumstances." (P. 399.) The so-called cases of cured deaf-dumbness, Dr. K. contends, are only cases in which the person had been taught to speak and understand what was said to him, by closely watching and imitating the motions of the lips of his instructor or other person addressing him. The hearing has in no case been in any considerable degree restored.

After the extensive analysis which we have given of the treatise of Dr. Kramer, it is hardly necessary for us to recommend it in the strongest terms to our readers. It is unquestionably, taken as a whole, the most valuable work we possess on the subject of diseases of the ear generally; and, after the labours of Itard and Deleau, it must be regarded as contributing more to the progress of acoustic surgery than any other modern publication.

Since the preceding pages were written, we have the satisfaction of knowing that a translation of the whole Treatise of Dr. Kramer has been undertaken by a scientific gentleman, already distinguished in his profession, and will be speedily in the hands of British surgeons. We would fain hope that the accomplished translator is destined to redeem the character of auricular surgery in this country.

